# Perioperative Patient Safety and Surgical Complications: A Comprehensive Review of Current Evidence and Improvement Strategies

**DOI:** 10.7759/cureus.99959

**Published:** 2025-12-23

**Authors:** Mohammed K Elbahi, Mohammed Fadlelmola Abdalla Mohamednour, Fatima S Mukhtar, Noha Rikabi, Sara I Elshafie

**Affiliations:** 1 Surgery, Faculty of Medicine, Al Neelain University, Khartoum, SDN; 2 Physiology, Faculty of Medicine, Al Neelain University, Khartoum, SDN; 3 Neurological Surgery, Al Neelain University, Khartoum, SDN; 4 Medicine and Surgery, Sudan International University, Khartoum , SDN; 5 Obstetrics &amp; Gynecology, Faculty of Medicine, University of Khartoum, Khartoum, SDN

**Keywords:** clinical guidelines, intraoperative safety, multidisciplinary care, perioperative safety, postoperative complications, postoperative monitoring, preoperative optimization, safety frameworks, surgical risk

## Abstract

Perioperative patient safety has become an increasingly urgent priority as global surgical demand rises and preventable postoperative complications continue to challenge healthcare systems. Despite major advances in anesthesia, surgical technique, and monitoring technologies, many adverse events still originate from modifiable, process-related factors occurring before, during, and immediately after surgery. These events commonly reflect an interaction between patient-specific physiological vulnerability, team performance, system reliability, and organizational culture.

This narrative review synthesizes contemporary evidence to examine specific perioperative interventions that have been associated with improved outcomes. Across the literature, early physiological deterioration is consistently identified as a major driver of preventable harm, underscoring the value of structured preoperative risk assessment and targeted optimization, as well as standardized intraoperative safety processes that support reliable team coordination. In the postoperative phase, structured surveillance systems and clearly defined escalation pathways are repeatedly linked to earlier recognition of deterioration and reduced respiratory and hemodynamic instability.

The review further highlights the role of teamwork, communication practices, and safety culture as enablers of reliable perioperative care. Institutions that implement structured communication routines, embed safety processes into daily workflows, and support a positive safety climate demonstrate more consistent application of evidence-based interventions and lower rates of preventable complications.

Overall, the findings support an integrated, system-level approach in which coordinated perioperative interventions rather than generalized vigilance alone link preoperative planning, intraoperative safety, and postoperative surveillance within a cohesive framework. Strengthening these coordinated processes provides a practical pathway for reducing avoidable harm and improving perioperative outcomes across diverse clinical settings.

## Introduction and background

Perioperative patient safety has become a central priority within modern surgical systems, as preventable postoperative complications continue to contribute significantly to morbidity despite major advances in anesthesia, surgical techniques, and monitoring technologies. Growing evidence demonstrates that many adverse events originate from modifiable factors distributed across the preoperative, intraoperative, and early postoperative phases, emphasizing the need for coordinated, evidence-based practices rather than isolated clinical interventions [1,2]. Complications such as respiratory deterioration and hemodynamic instability remain particularly common when physiological vulnerabilities are insufficiently recognized or optimized before surgery, underscoring the importance of structured preoperative assessment pathways [3].

Current literature increasingly highlights that perioperative safety is shaped by system-level dynamics, including communication quality, interdisciplinary coordination, and organizational culture. Studies assessing perioperative workflows reveal that communication lapses, inconsistent adherence to recommended practices, and variability in team performance directly influence surgical reliability and the likelihood of preventable harm [4,5]. These insights support the shift from attributing complications to single failures toward recognizing their cumulative nature, where small process deviations across care phases increase susceptibility to postoperative deterioration [6].

Recent reviews consistently identify opportunities to reduce preventable complications through strengthened preoperative risk evaluation, reliable intraoperative safety processes, and structured early postoperative surveillance. Standardized perioperative bundles and guideline-based care pathways have been associated with reductions in early respiratory events, hemodynamic instability, and unplanned escalation of care when applied consistently across institutions [2,6]. Sustained improvement efforts further highlight that long-term progress depends on continuous monitoring, iterative feedback, and organizational commitment to embedding safety principles into routine workflows [7].

Despite progress, perioperative outcomes continue to vary across healthcare settings. Differences in institutional culture, resource allocation, and implementation fidelity contribute to disparities in complication rates, reinforcing the need for harmonized evaluation frameworks and structured benchmarking. Reviews focusing on postoperative complications also point to persistent challenges associated with comorbidity burden, inconsistent surveillance, and incomplete perioperative planning, particularly among high-risk patients [8]. To address these gaps, consensus-based safety indicators derived through Delphi methodologies have been proposed to guide systematic assessment and support targeted improvement initiatives [9].

Taken together, the emerging evidence underscores the need for an integrated, system-level approach to perioperative safety, one that aligns preoperative optimization, intraoperative reliability, postoperative vigilance, and institutional safety culture. This review synthesizes findings from nine contemporary publications to provide a comprehensive overview of perioperative patient safety and postoperative complications, outlining practical strategies capable of improving outcomes across diverse clinical environments. Unlike prior reviews that primarily address isolated perioperative phases or specific interventions, this review provides an integrated, system-level synthesis linking preoperative risk assessment, intraoperative human factors, and postoperative surveillance within a coordinated perioperative safety framework.

## Review

Methodology

This narrative review was conducted using a structured and rigorous approach guided by the principles of the Scale for the Assessment of Narrative Review Articles (SANRA). The methodology was designed to ensure comprehensive coverage of contemporary evidence on perioperative patient safety, postoperative complications, and improvement strategies. The process included a systematic literature search, clearly defined eligibility criteria, structured data extraction, and a quality assessment phase to ensure the reliability and relevance of the synthesized findings.

Search Strategy

A structured and comprehensive search was conducted to identify relevant literature addressing perioperative patient safety, postoperative complications, and improvement strategies. Major academic databases, including PubMed, Scopus, Web of Science, and Google Scholar, were searched using combinations of relevant keywords and Medical Subject Headings (MeSH) related to perioperative care, patient safety, postoperative complications, safety culture, and system-level improvement approaches. Boolean operators (AND, OR) were applied to refine the search. In addition, reference lists of selected articles were manually screened to identify further relevant publications.

Inclusion and Exclusion Criteria

Eligibility criteria were established to ensure that only high-quality and relevant evidence was included. Studies were eligible if they focused on perioperative safety frameworks, patterns of postoperative complications, perioperative risk factors, or evidence-based strategies aimed at improving patient safety across the surgical continuum. The literature selection prioritized recent and clinically relevant publications, while also including seminal or widely cited works where necessary to provide conceptual context. Peer-reviewed articles, narrative and systematic reviews, clinical guidelines, and well-designed observational studies were considered. Publications not directly relevant to perioperative patient safety, those focusing exclusively on pediatric populations or highly subspecialized outcomes without broader applicability, and non-English language publications were excluded.

Data Extraction

A structured data extraction process was used to collect key information from selected studies. Extracted data included study objectives, methodology, population characteristics, perioperative risk factors, patterns of postoperative complications, safety interventions, and major outcomes. Information related to human factors, communication quality, institutional safety culture, and guideline adherence was also collected due to their relevance in the perioperative safety domain. A standardized extraction template was used to ensure uniformity and minimize bias.

Quality Assessment

To ensure the credibility and validity of the included studies, each article underwent a methodological quality assessment using established critical appraisal tools. The Joanna Briggs Institute (JBI) critical appraisal instruments were applied according to study design. This assessment allowed for the exclusion of low-quality evidence and strengthened the reliability of findings synthesized in the review. The evaluation process focused on methodological clarity, completeness of reporting, accuracy of outcome measures, and relevance to perioperative safety.

Synthesis of Findings

A narrative synthesis approach was used to integrate data extracted from the eligible studies. Findings were organized thematically into major domains, including perioperative safety frameworks, preoperative optimization, intraoperative human factors, postoperative complications, and system-level strategies for improvement. Themes, patterns, and recurring challenges were identified to provide a cohesive understanding of current evidence. This structured synthesis allowed for clear narrative progression, highlighting both advances in perioperative safety and persistent gaps requiring further investigation.

By adhering to SANRA standards and applying a meticulous, systematic methodology, this review provides a comprehensive and analytically rich overview of perioperative patient safety and postoperative complication prevention.

Perioperative safety frameworks and current recommendations

Contemporary perioperative safety frameworks have increasingly shifted toward structured, multidisciplinary systems designed to anticipate and prevent harm across all stages of surgical care. Recent reviews emphasize that effective frameworks depend on integrating clear clinical guidelines with standardized communication practices and continuous quality-improvement mechanisms that reduce intraoperative and postoperative risks [1,10]. These frameworks recognize that perioperative safety is shaped not only by clinical decisions but also by organizational culture, teamwork reliability, and consistent adherence to evidence-based protocols [5,11].

A major development in recent perioperative practice is the movement toward harmonizing safety guidelines across institutions. Evidence shows that implementing standardized perioperative bundles covering structured risk assessment, early detection of physiological deterioration, and coordinated postoperative surveillance has been associated with measurable reductions in complication rates when consistently applied [6]. These bundles commonly highlight essential components such as preoperative optimization, airway and anesthesia safety, sterile technique, fluid and hemodynamic management, and prevention of early respiratory or cardiovascular instability.

Systems-level evaluations further underscore the importance of embedding patient-centered and team-based principles within perioperative pathways. Improved coordination during handovers, clearer distribution of roles, and more reliable transfer of risk-related information between surgical, anesthesia, and nursing teams have been linked to enhanced safety and greater adherence to recommended practices [4,12]. Studies examining perioperative safety culture consistently show that institutions with stronger safety climate demonstrate lower preventable complication rates and more predictable application of perioperative standards [5,13].

Long-term sustainability also remains a defining feature of effective perioperative frameworks. Evidence indicates that durable improvement requires hospitals to employ continuous monitoring systems, structured feedback loops, and iterative learning cycles rather than isolated audits or short-term corrective actions [7]. These processes strengthen system reliability, reduce unwarranted variation in practice, and create the foundation for sustained safety performance.

Preoperative assessment and optimization

Preoperative assessment is a high-yield point for preventing downstream complications because it determines how well patient-specific physiological vulnerabilities are identified, communicated, and actively mitigated before surgery. Rather than emphasizing “assessment” in general terms, recent evidence supports the practical value of structured preoperative evaluation pathways (e.g., standardized pre-anesthesia assessment and risk-directed optimization) in reducing early perioperative respiratory and hemodynamic instability and preventing unplanned escalation of care when applied consistently [3,6].

A second actionable element is risk stratification that directly informs perioperative planning, including the level of intraoperative monitoring and the appropriate postoperative destination (ward vs. higher-acuity care). Contemporary risk-assessment literature highlights that structured risk stratification is most meaningful when it results in a clear plan, for example, identifying high-risk patients for closer monitoring and early escalation triggers [14].

Finally, optimization should be described in terms of what is being optimized and why it matters. Guideline-oriented perioperative frameworks and implementation studies emphasize that improvements are more likely when optimization steps are standardized, communicated across the multidisciplinary team, and embedded into routine workflows rather than left to individual practice variation [4,6]. Patient engagement (education, expectation management, and adherence to perioperative instructions) also supports safer recovery trajectories and reduces avoidable postoperative deterioration (Table 1) [5].

**Table 1:** Preoperative interventions and their outcome-relevant intent

Specific preoperative intervention	What it operationalizes	Reference	Outcome-relevant intent (what it helps prevent)
Structured preoperative evaluation pathway (standardized clinic/assessment process)	Early detection + targeted optimization of risk	[3,6]	Early respiratory/hemodynamic instability; unplanned escalation of care
Risk stratification used to determine monitoring intensity and postoperative destination	Risk-informed planning (not risk scoring alone)	[6,14]	Failure-to-rescue scenarios; delayed escalation
Standardized preoperative optimization embedded in guidelines/workflows	Consistent implementation across teams	[4,6]	Variation-driven preventable complications
Patient education and engagement (expectations, instructions, adherence)	Shared preparedness and compliance	[5]	Avoidable early deterioration linked to nonadherence/communication gaps

Intraoperative safety principles and human factors

The intraoperative phase represents a critical point at which system reliability and team performance directly influence patient outcomes. Evidence from safety science and perioperative literature indicates that improvements in intraoperative safety are most effective when specific, standardized interventions are used to reduce variability and support shared situational awareness rather than relying on individual vigilance alone [10,11].

One of the most consistently studied interventions is the use of structured safety processes, such as surgical safety checklists and pre-incision briefings. While checklist implementation alone does not uniformly reduce adverse events, studies and guideline-based evaluations show that checklists are most beneficial when integrated into routine workflows and combined with active team communication, role clarity, and leadership engagement [10,15]. Under these conditions, checklists function as tools to prompt critical steps, surface latent risks, and align the surgical team before irreversible actions occur.

Human factor research further demonstrates that communication quality, teamwork, and shared mental models are central determinants of intraoperative reliability. Breakdowns in information transfer, unclear task allocation, and hierarchical barriers have been repeatedly associated with preventable intraoperative errors and downstream complications [11]. Conversely, structured communication routines such as team briefings and closed-loop communication support early recognition of evolving risks and timely corrective action.

Importantly, sustained intraoperative safety improvement depends on system-level reinforcement, including training, feedback mechanisms, and a supportive safety culture. Institutions that embed human factors principles into daily practice and reinforce expected behaviors demonstrate more consistent adherence to intraoperative safety processes and reduced variability in performance [4,5]. These findings emphasize that intraoperative safety is not achieved through isolated tools but through coordinated interventions that strengthen team reliability in real clinical environments (Table 2).

**Table 2:** Intraoperative interventions linked to outcome-relevant mechanisms

Specific intraoperative intervention	What it operationalizes	Reference	Outcome-relevant intent
Surgical safety checklist integrated with active team communication	Standardization + risk prompting	[10,15]	Prevention of omitted safety steps; reduced intraoperative errors
Pre-incision briefings and structured team communication	Shared situational awareness	[5,11]	Early identification of intraoperative risks
Closed-loop communication and clear role allocation	Reduction of communication failures	[11]	Fewer preventable intraoperative incidents
Embedding human factors training into routine practice	System reliability over individual vigilance	[4,5]	Reduced variability in intraoperative performance

Postoperative complications: patterns and contributing factors

The immediate postoperative period is a high-risk phase during which physiological deterioration frequently precedes major adverse outcomes. Evidence from perioperative safety and postoperative care literature consistently shows that many serious complications are not sudden events but rather evolve over time and are preceded by detectable abnormalities in vital signs, respiratory status, or mental state [16]. Consequently, postoperative safety is strongly influenced by the effectiveness of surveillance systems and escalation pathways, rather than by vigilance alone.

One of the most outcome-relevant interventions in this phase is the use of structured postoperative monitoring frameworks, including early warning scores and protocolized response systems. Observational studies and quality-focused reviews indicate that such frameworks support earlier recognition of deterioration and reduce delays in escalation, which are key contributors to failure-to-rescue events [16,17]. These systems are most effective when escalation thresholds are clearly defined and supported by institutional policies that empower timely clinical response.

Another critical determinant of postoperative outcomes is the quality of handover and continuity of care between intraoperative and postoperative teams. Inconsistent information transfer regarding intraoperative events, residual risks, or monitoring priorities has been repeatedly linked to delayed recognition of complications and unplanned critical-care admission [12,18]. Structured handover processes improve the reliability of information transfer and help ensure that postoperative monitoring is aligned with patient-specific risk.

Importantly, postoperative surveillance should be viewed as the final component of an integrated perioperative safety pathway. When postoperative monitoring is disconnected from preoperative risk stratification and intraoperative events, opportunities for early intervention are often missed. Evidence suggests that coordinated surveillance strategies aligned with earlier perioperative planning are associated with fewer preventable complications and improved management of early physiological deterioration [7,16] (Table 3).

**Table 3:** Postoperative surveillance interventions linked to outcome-relevant mechanisms

Specific postoperative intervention	What it operationalizes	Reference	Outcome-relevant intent
Early warning score–based monitoring	Early detection of physiological deterioration	[16,17]	Reduced failure-to-rescue events
Protocolized escalation pathways	Timely response to abnormal findings	[16]	Fewer unplanned ICU admissions
Structured postoperative handover	Continuity of risk-related information	[12,18]	Earlier recognition of complications
Surveillance aligned with pre/intraoperative risk	Integrated perioperative planning	[7,16]	Reduced preventable postoperative harm

Discussion

Improving perioperative patient safety requires recognizing that complications rarely arise from isolated clinical errors; instead, they typically emerge from the interplay between patient-related vulnerabilities, system-level gaps, and inconsistent adherence to recommended practices across the surgical continuum. Recent evidence emphasizes that perioperative safety cannot be achieved through clinical interventions alone but depends on coordinated processes spanning preoperative risk identification, intraoperative reliability, and postoperative vigilance [5].

A consistent theme across the literature is that early physiological deterioration remains one of the strongest predictors of postoperative complications. This deterioration commonly occurs during the immediate postoperative period, when patients are highly susceptible to respiratory and hemodynamic instability. Such events often reflect inadequate preoperative optimization, incomplete recognition of baseline risk factors, or unresolved intraoperative stress. These insights reinforce the need for comprehensive preoperative assessment pathways that integrate physiological evaluation, risk modification, and patient engagement to enhance preparedness and adherence to perioperative instructions.

The intraoperative phase continues to represent a critical point of vulnerability. While advances in surgical technology and monitoring have mitigated certain risks, human factors particularly communication breakdowns, inconsistent application of safety checklists, and variability in team coordination remain major contributors to preventable harm. Institutions with stronger safety culture demonstrate better adherence to perioperative processes, highlighting the influence of organizational climate on surgical performance. Strengthening intraoperative safety therefore requires not only technical precision but also reliable teamwork, structured communication routines, and an environment that supports speaking up and maintaining shared mental models.

Postoperative complications often reflect the cumulative effect of the entire perioperative journey. Gaps in monitoring, delayed recognition of early warning signs, and inconsistent escalation practices contribute significantly to unplanned critical-care transfers, infections, and cardiopulmonary instability. Evidence supports the use of structured postoperative surveillance frameworks with clear escalation thresholds to improve early detection and timely management of deterioration. However, variability in practice across institutions underscores the need for standardized pathways that ensure continuity between preoperative planning, intraoperative performance, and postoperative care.

Long-term improvement in perioperative safety is strongly linked to organizational commitment. While short-term audits or standalone safety initiatives can produce temporary benefits, sustainable progress requires continuous monitoring, iterative learning cycles, and feedback mechanisms that allow teams to evaluate performance over time. Harmonized national and institutional guidelines further strengthen these efforts by establishing shared expectations, reducing practice variability, and enabling benchmarking across surgical systems.

Overall, recent evidence underscores that high-quality perioperative care relies on a coordinated, system-level approach in which standardized guidelines, effective interdisciplinary communication, and supportive organizational culture function together to reduce preventable harm. Integrating structured preoperative risk screening, physiologic optimization, and patient engagement with reliable intraoperative practices and consistent postoperative surveillance forms the foundation for minimizing early deterioration and achieving safer surgical outcomes. These combined principles provide a robust framework for developing comprehensive perioperative safety pathways capable of sustainably improving patient outcomes across diverse clinical environments.

## Conclusions

Perioperative patient safety is a multidimensional, system-dependent challenge influenced by patient risk profiles, organizational processes, and the consistency of clinical practice. Current evidence shows that most preventable postoperative complications arise not from isolated errors but from cumulative gaps in preoperative risk assessment, intraoperative coordination, and early postoperative surveillance. Strengthening perioperative care therefore requires integrated, standardized pathways that combine thorough preoperative optimization, reliable intraoperative teamwork, and vigilant postoperative monitoring with clear escalation triggers. Sustained improvement depends on continuous evaluation, iterative learning, and institutional cultures that prioritize safety and adherence to evidence-based practices. Embedding these components within cohesive perioperative frameworks offers an effective strategy for reducing preventable harm and improving surgical outcomes.
